# Rezidivierendes DMEK-Versagen

**DOI:** 10.1007/s00347-020-01184-5

**Published:** 2020-07-29

**Authors:** C. Matar, B. Seitz, L. Daas

**Affiliations:** Klinik für Augenheilkunde, Universitätsklinikum des Saarlandes UKS, Kirrberger Str. 100, 66424 Homburg/Saar, Deutschland

**Keywords:** Descemet Membrane Endothelial Keratoplasty, Transplantatversagen, Herpetische Uveitis, Endotheliitis, Zystoides Makulaödem, Descemet Membrane Endothelial Keratoplasty, Graft failure, Herpetic uveitis, Endotheliitis, Cystoid macular edema

## Abstract

Wir berichten über einen Fuchs-Endotheldystrophie-Patienten mit drei „Descemet Membrane Endothelial Keratoplasty“ (DMEK) bei rezidierendem Transplantatversagen mit Intraokulardruckerhöhung und cystoides Makulaödem. Bei der dritten DMEK wurde Herpes im Vorderkammeraspirat nachgewiesen und eine adäquate Therapie eingeleitet. Bei der 6 Monaten-Kontrolle blieb die Hornhaut klar, der Visus betrug 0,8, der Intraokulardruck lag im Normbereich und das Makulaödem bildete sich zurück. Entweder wurde eine latente Herpes simplex Virus(HSV)-Infektion des Patienten reaktiviert oder eine befallene Spender-Lamelle transplantiert mit Spender-zu-Wirt-zu-Spender „Ping-Pong“ Übertragung.

## Abstract (en)

We report a Patient with Fuchs dystrophy who underwent three Descemet Membrane Endothelial Keratoplasties (DMEK) caused by recurrent graft failure with raise in intraocular pressure and cystoid macular edema. At the third DMEK, herpes was detected in the anterior chamber tap and an adequate therapy was initiated. At the 6 months follow-up the cornea remained clear, visual acuity was 0.8, intraocular pressure was within normal range and macular edema regressed completely. Either a latent Herpes simplex Virus (HSV) infection of the patient was reactivated or an infected donor lamella was transplanted with donor-to-host-to-donor ping-pong transmission.

## Anamnese

**Figure 1:**
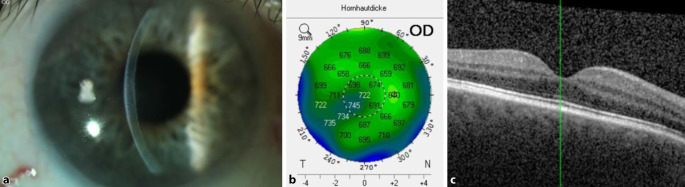

Ein 66-jähriger pseudophaker Patient wurde bei Fuchs-Endotheldystrophie in unsere Klinik überwiesen. Anamnestisch gab er eine progressive Sehverschlechterung mit vermehrter Blendung am rechten Auge ohne bekannte vorausgegangene ophthalmologische oder systemrelevante Erkrankung an. Die Sehkraft war v. a. in den Morgenstunden reduziert und besserte sich allmählich im Laufe des Tages. Die bestkorrigierte Sehschärfe (BKSS) am rechten und linken Auge betrug 0,4 und 0,8, der Intraokulardruck (IOD) 15 und 17 mm Hg, die zentrale Hornhautdicke 722 und 615 μm. Fundoskopisch zeigten sich beidseits vitale, randscharfe Papillen ohne pathologische Exkavation. Der Makula-OCT-Befund war beidseits unauffällig (Abb. 1). Am rechten Auge zeigte sich eine Hornhautdekompensation mit epithelialen Bullae ohne Hornhautnarben (Abb. 1) und am linken Auge eine kompensierte Hornhaut. Wir führten am rechten Auge eine komplikationslose „Descemet membrane endothelial keratoplasty“ (DMEK) mit 20 % Schwefelhexafluoride(SF6)-Gastamponade durch. Die Endothelzelldichte (EZD) des Transplantates betrug 2200 Zellen/mm^2^ (Z/mm^2^). Sechs Wochen postoperativ zeigten sich eine BKSS von 0,8 und eine Erhöhung des Intraokulardrucks (IOD) auf 24 mm Hg. Eine lokale drucksenkende Therapie wurde mit lokalen β‑Blocker-Tropfen (Timolol® 0,5 %) 2‑mal pro Tag angesetzt und bei Verdacht auf Steroidresponse Prednisolonacetat 5 mal pro Tag auf Loteprednol etabonat 5‑mal pro Tag umgestellt. Im weiteren Verlauf zeigte sich eine unzureichende Drucksenkung mit IOD von 23 mm Hg. Deswegen wurde die antiglaukomatöse Therapie auf ein Kombipräparat mit β‑Blocker und α2-Adrenozeptor-Agonist (Combigan®) umgestellt. Hierunter normalisierte sich der IOD. Das Transplantat war stets komplett anliegend; 24 Monate nach der Operation war der IOD am rechten Auge medikamentös gut reguliert, die bestkorrigierte Sehschärfe betrug 0,6, die EZD 1671 Z/mm^2^, die Hornhautdicke 614 μm.

## Klinischer Befund

**Figure 2:**
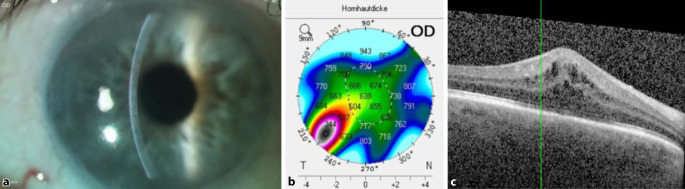

Der Patient wurde 28 Monate nach DMEK erneut überwiesen. Die BKSS betrug 0,05 und der IOD 14 mm Hg unter Loteprednol 1‑mal pro Tag. Es zeigte sich eine Hornhaut-Endothel-Epithel-Dekompensation (HEED) mit epithelialen Bullae, Descemet-Falten und einzelnen Zellen und Tyndall + in der Vorderkammer ohne sichtbare retrokorneale Beschläge. Die zentrale Hornhautdicke (CCT) betrug 638 μm und die EZD 700 Z/mm^2^. In der Makula-OCT zeigte sich ein zystoides Makulaödem (CMÖ) (Abb. 2). Bei initialem Verdacht auf endotheliale Transplantatabstoßungsreaktion wurde die topische Steroidtherapie auf stündlich erhöht. Bei ausbleibender Befundverbesserung nach 2 Wochen und Verdacht auf sekundäres Transplantatversagen führten wir eine Re-DMEK (TPL-Durchmesser 7,5 mm) mit einer simultanen parabulbären Triamcinolonacetonid-Eingabe (40 mg) bei Verdacht auf Late-onset-Irvine-Gass-Syndrom nach DMEK durch. Dabei kam es 2 Monate danach zu einer kompletten Regression des Vorderkammerreizzustands, der Visus stieg auf 0,8, der IOD betrug 21 mm Hg und die CCT 582 μm. In der Makula-OCT zeigte sich jedoch nur eine leichte Befundbesserung mit Restödem.

**Figure 3:**
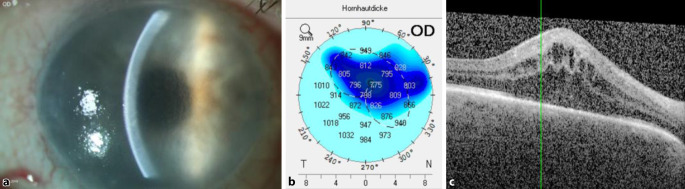

Sechs Monate danach zeigte sich eine erneute Befundverschlechterung mit erneuter kompletter Hornhautdekompensation HEED und epithelialen Bullae unter topischen Steroiden 3‑mal pro Tag im Bereich der zentralen 7,5 mm und der Wirtshornhaut. Die CCT betrug 788 μm und der IOD 18 mm Hg unter oben genannter lokaler antiglaukomatöser Therapie. Klinisch zeigte sich ein milder Vorderkammerreizzustand ohne Irisretroillumination. Die DMEK-Lamelle war zirkulär anliegend, und das CMÖ nahm zu (Abb. 3). Eine intravitreale Dexamethason-Implantation wurde durchgeführt. Zwei Wochen danach erfolgte eine Re-Re-DMEK mit diagnostischer Vorderkammerpunktion. Dabei war der Polymerasekettenreaktion(PCR)-Befund positiv für Herpes simplex Virus (HSV) Typ I und negativ für CMV, VZV und EBV.

## Diagnose

Rezidivierendes Transplantatversagen bei herpetischer Uveitis mit Endotheliitis und Trabekulitis.

## Therapie und Verlauf

**Figure 4:**
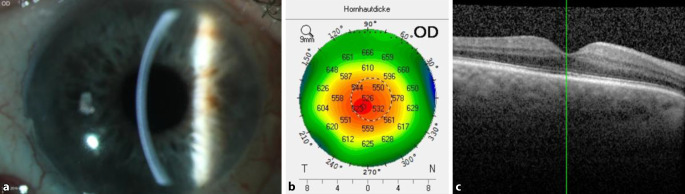

Eine systemische und lokale antiherpetische und Cortison-Therapie mit Aciclovir 500 mg i.v. 3‑mal pro Tag, Prednisolon 150 mg i.v. pro Tag, Ganciclovir Augengel 5‑mal pro Tag sowie Loteprednol etabonat-Augentropfen stündlich wurde am ersten postoperativen Tag nach der letzten DMEK eingeleitet. Die systemische Therapie erfolgte über 3 Tage und wurde dann oralisiert und langsam ausgeschlichen (Aciclovir 400 mg 5‑mal pro Tag über 6 Wochen dann 2‑mal pro Tag 400 mg über 1 Jahr, Prednisolon 100 mg alle 4 Tage um 20 mg reduziert). Die lokale Entlassungstherapie bestand aus Ganciclovir-Augengel 5‑mal pro Tag, nach 6 Wochen auf 3‑mal pro Tag reduziert und bei 1‑mal pro Tag lebenslang belassen, Loteprednol etabonat 5‑mal pro Tag, alle 8 Wochen um 1 Tropfen weniger und bei 2‑mal pro Tag lebenslang belassen, Brimonidin-Augentropfen 2‑mal pro Tag. Bei der letzten ambulanten Verlaufskontrolle (6 Monaten postoperativ) zeigte sich am rechten Auge die Hornhaut glatt und klar sowohl zentral als auch peripher, das Descemet/Endotheltransplantat war klinisch und in der VAA-OCT zirkulär anliegend. Der bestkorrigierte Visus betrug 0,8, die tomographische zentrale Hornhautdicke betrug 526 μm, der IOD lag bei 16 mm Hg applanatorisch unter lokaler drucksenkender Therapie, die Vorderkammer war reizfrei, und es fanden sich weder retrokorneale Beschläge noch ein CMÖ (Abb. 4**)**.

## Diskussion

Zu dem klassischen klinischen Bild einer herpetischen Uveitis und Endotheliitis gehören: Hornhautödem, Descemet-Falten, retrokorneale Beschläge, milder Vorderkammerreizzustand und Augeninnendruckerhöhung im Sinne einer Trabekulitis bei viralem Befall des Trabekelmaschenwerks. Wenn nicht rechtzeitig erkannt und behandelt, kann ein irreversibler Hornhautendothel- und sogar Sehverlust drohen [10, 11].

Zarei-Ghanavati et al. berichteten 2015 zum ersten Mal über eine Herpes-Reaktivierung nach DMEK kombiniert mit Kataraktoperation (New-Triple-DMEK) bei vorbekannter anteriorer Uveitis. Dabei zeigten sich am dritten postoperativen Tag ein fokales Hornhautödem mit Transplantatdehiszenz und am fünften Tag eine partielle Transplantatdehiszenz, infolgedessen ein Re-Bubbling durchgeführt wurde, jedoch ohne Befundbesserung. Am siebten postoperativen Tag zeigten sich diffuse retrokorneale Präzipitate *sowohl auf der Wirts- als auch Spenderhornhaut*. Eine HSV-1-Infektion wurde mittels Vorderkammerpunktion und PCR bestätigt und eine systemische und lokale antivirale Therapie eingeleitet. Zwei Tage später zeigte sich eine erhebliche Befundbesserung mit klarer Hornhaut und kompletter Regression der Präzipitate [15].

In einer Beobachtung im Tiermodell spielt die vorderkammerassoziierte Immunabweichung (ACAID) bei dem vermuteten ätiopathologischen Mechanismus der Endotheliitis eine wesentliche Rolle. Dadurch steigt die Schwelle für eine zellvermittelte Immunantwort. In unserem Fall ist ein Vorderkammerreizzustand (Zellen, Tyndall und retrokorneale Präzipitate) kaum zu sehen, allerdings vermehrt sich gleichzeitig aktiv die Viruslast in den Endothelzellen und führt zum Transplantatversagen [16]. Die Viruslast in den Endothelzellen wurde jedoch in unserer Studie nicht untersucht.

Der Einsatz eines routinemäßigen Screenings gegen Herpes in der Hornhautbank hat sich in der klinischen Anwendung bisher nicht bewährt. Gründe dafür sind zum einen die „operationelle Latenz“, d. h. die Abwesenheit von infektiöser Viruslast im zu transplantierenden Hornhautendothel zum Zeitpunkt der Explantation des Spenders, mit zeitversetzter Aktivierung nach der Transplantation [3]. Zum anderen ist die PCR-Sensibilität gegenüber Herpes beschränkt. In einer wesentlichen Arbeit untersuchten Remeijer et al. exzidierte Hornhäute von 83 Patienten mit bekannter Herpeskeratitis (Gruppe 1) und 367 Patienten ohne Herpeskeratitis (Gruppe 2) anhand PCR. Dabei zeigte sich interessanterweise eine Detektionsrate von nur 48 % in Gruppe 1 vs. 4 % in Gruppe 2 [9].

Stavridis et al. berichteten 2012 über eine rezidivierende „primäre Transplantatinsuffizienz“ jedoch nach perforierender Hornhauttransplantation. Dabei zeigte sich retrospektiv in der HSV-Immunhistochemie und PCR-Analyse ein positiver Nachweis in dem ersten und zweiten dekompensierten Transplantat bei negativem Nachweis in der exzidierten Wirtshornhaut, was auf ein kontaminiertes Transplantat und eine Spender-zu-Wirt-zu-Spender-„Pingpong“-Herpes-Übertragung hinwies [14]. Da im vorliegenden Fall keine Untersuchung der Wirtshornhaut erfolgte, konnte nicht sicher differenziert werden, ob das HSV vom Spender oder möglicherweise doch vom Wirt stammte.

Man spricht von primärem Transplantatversagen, wenn die Hornhaut nach der Hornhauttransplantation zu keinem Zeitpunkt aufklart. Bei sekundärem Transplantatversagen klart die Hornhaut initial auf, und der Visus steigt an, bevor es später zu einer Endotheldekompensation mit Sehschärfeverlust und Hornhautödem kommt [5, 12]. Als häufigste Ursache eines Transplantatversagens in der Frühphase nach DMEK gilt die endotheliale Immunreaktion mit einer Inzidenz von 1–5 % [7]. Entscheidend dabei ist, die topische Steroidapplikation sehr langsam auszuschleichen, um das Risiko einer Immunreaktion zu minimieren [4]. Weniger häufige Ursachen eines sekundären Versagens stellen virale Uveitiden dar. Dabei können herpetische anteriore Uveitiden mit HSV, VZV, CMV und EBV zu einem Versagen führen [13], wobei der Vorderkammerreizzustand gering sein oder fehlen kann.

Bezüglich des CMÖ nach DMEK berichten Bachmann et al. über eine Inzidenz von ca. 10 %.

Diese Komplikation lässt sich allerdings durch die prophylaktische hochfrequente Anwendung von topischen Steroiden während der ersten postoperativen Woche signifikant reduzieren [2]. Ähnlich zeigten Kocaba et al. in einer Gruppe von 80 Patienten eine gesamte Inzidenz (DMEK/New-Triple-DMEK) von 13,8 % (DMEK 8,1 %, New-Triple 18,6 %). Laut Autorin waren jedoch weder Risikofaktoren für das Auftreten eines CMÖs nach DMEK zu identifizieren noch ein dauerhafter Sehschärfeverlust zu beklagen [8]. Seltener tritt ein CMÖ als sekundäre Komplikation bei viraler anteriorer Uveitis mit HSV mit einer Inzidenz von 8 % auf [6]. In beiden Fällen besteht die Behandlung primär aus Steroiden mit zusätzlicher antiviraler Therapie bei Herpesinfektion.

Unsere Arbeitsgruppe berichtete 2018 über gute Ergebnisse nach New-Triple-DMEK unter systemischer antiviraler Therapie mit Aciclovir über 1 Jahr bei Endotheldekompensation wegen rezidivierender herpetischer Endotheliitis [1].

In unserem Fall wurde entweder eine latente HSV-Infektion des Patienten durch die DMEK reaktiviert oder eine befallene Spenderlamelle transplantiert. Die Augeninnendruckerhöhung konnte differenzialdiagnostisch auf 2 Mechanismen zurückgeführt werden: entweder direkte Folge einer herpetischen Trabekulitis und/oder aufgrund eines Steroidresponse.

## Fazit für die Praxis

Bei folgender klinischer Konstellation nach DMEK sollte differenzialdiagnostisch stets an eine herpetische Genese gedacht werden (HSV, VZV, CMV, EBV) und die adäquate Therapie unverzögert eingeleitet werden:

- rezidivierendes DMEK-Versagen ohne technischer Grund,
- Dekompensation sowohl im Bereich des Transplantates als auch der peripheren Wirtshornhaut bei Fuchs-Endotheldystrophie als Grunderkrankung,
- milder Vorderkammerreizzustand und IOD-Erhöhung trotz fehlender retrokornealer Beschläge,
- ggf. CMÖ.
